# Diagnosis of prostate cancer with magnetic resonance imaging in men treated with 5-alpha-reductase inhibitors

**DOI:** 10.1007/s00345-023-04634-2

**Published:** 2023-10-03

**Authors:** Ugo G. Falagario, Anna Lantz, Ivan Jambor, Gian Maria Busetto, Carlo Bettocchi, Marco Finati, Anna Ricapito, Stefano Luzzago, Matteo Ferro, Gennaro Musi, Angelo Totaro, Marco Racioppi, Umberto Carbonara, Enrico Checcucci, Matteo Manfredi, Damiano D’Aietti, Antonio Benito Porcaro, Tobias Nordström, Lars Björnebo, Marco Oderda, Francesco Soria, Pekka Taimen, Hannu J. Aronen, Ileana Montoya Perez, Otto Ettala, Michele Marchioni, Giuseppe Simone, Mariaconsiglia Ferriero, Aldo Brassetti, Luigi Napolitano, Luca Carmignani, Claudia Signorini, Andrea Conti, Giuseppe Ludovico, Marcello Scarcia, Carlo Trombetta, Francesco Claps, Fabio Traunero, Emanuele Montanari, Luca Boeri, Martina Maggi, Francesco Del Giudice, Pierluigi Bove, Valerio Forte, Vincenzo Ficarra, Marta Rossanese, Giuseppe Mucciardi, Vincenzo Pagliarulo, Alessandro Tafuri, Vincenzo Mirone, Luigi Schips, Alessandro Antonelli, Paolo Gontero, Luigi Cormio, Alessandro Sciarra, Francesco Porpiglia, PierFrancesco Bassi, Pasquale Ditonno, Peter J. Boström, Emanuele Messina, Valeria Panebianco, Ottavio De Cobelli, Giuseppe Carrieri, The PROMOD Study Group

**Affiliations:** 1 Unit of Urology, Department of Molecular Medicine and Surgery, Karolinska Institutet, Stockholm, Sweden; 2 Department of Urology and Organ Transplantation, University of Foggia, Foggia, Italy; 3 Department of Medical Epidemiology and Biostatistics, Karolinska Institutet, Stockholm, Sweden; 4 Department of Radiology, University of Turku, Turku, Finland; 5 Medical Imaging Centre of Southwest Finland, Turku University Hospital, Turku, Finland; 6 Department of Urology, IEO European Institute of Oncology, IRCCS, Milan, Italy; 7 Department of Oncology and Hemato-Oncology, Università Degli Studi Di Milano, Milan, Italy; 8 Department of Urology, Catholic University Medical School “A. Gemelli” Hospital, Rome, Italy; 9 Department of Urology, Andrology and Kidney Transplantation, University of Bari, Bari, Italy; 10 Department of Urology, Azienda Ospedaliera Universitaria “San Luigi Gonzaga”, University of Turin, Turin, Italy; 11 UOC Urologia, Azienda Ospedaliera Universitaria Integrata Di Verona, Verona, Italy; 12 Department of Surgical Sciences, Città Della Salute E Della Scienza Di Torino, Molinette Hospital, Turin, Italy; 13 Institute of Biomedicine, University of Turku, Turku, Finland; 14 Department of Pathology, Turku University Hospital, Turku, Finland; 15 Department of Urology, University of Turku, Turku, Finland; 16 Turku University Hospital, Turku, Finland; 17 Department of Urology, Università “G.d’Annunzio”, Chieti-Pescara, Italy; 18 Department of Oncologic Urology, IRCCS “Regina Elena” National Cancer Institute of Rome, Rome, Italy; 19 Department of Urology, University of Naples Federico II, Naples, Italy; 20 IRCCS Policlinico San Donato, Milan, Italy; 21 Department of Urology, Ente Ecclesiastico Miulli, Acquaviva Delle Fonti, Italy; 22 Clinica Urologica Di Trieste, Trieste, Italy; 23 Department of Urology, IRCCS Foundation Ca’ Granda-Maggiore Policlinico Hospital, Milan, Italy; 24 Department of Maternal Infant and Urological Sciences, Sapienza Rome University, Rome, Italy; 25 Department of Urology, San Carlo Di Nancy Hospital, Rome, Italy; 26 Department of Urology, University of Messina, Messina, Italy; 27 Department of Urology, Vito Fazzi Hospital, Lecce, Italy; 28 Department of Urology, Ospedale L. Bonomo, Andria, Italy; 29 Department of Radiological Sciences, Oncology and Pathology, Sapienza University/Policlinico Umberto I, Rome, Italy

**Keywords:** Magnetic resonance imaging, Prostate cancer, 5-Alpha-reductase inhibitors

## Abstract

**Purpose:**

The primary aim of this study was to evaluate if exposure to 5-alpha-reductase inhibitors (5-ARIs) modifies the effect of MRI for the diagnosis of clinically significant Prostate Cancer (csPCa) (ISUP Gleason grade ≥ 2).

**Methods:**

This study is a multicenter cohort study including patients undergoing prostate biopsy and MRI at 24 institutions between 2013 and 2022. Multivariable analysis predicting csPCa with an interaction term between 5-ARIs and PIRADS score was performed. Sensitivity, specificity, and negative (NPV) and positive (PPV) predictive values of MRI were compared in treated and untreated patients.

**Results:**

705 patients (9%) were treated with 5-ARIs [median age 69 years, Interquartile range (IQR): 65, 73; median PSA 6.3 ng/ml, IQR 4.0, 9.0; median prostate volume 53 ml, IQR 40, 72] and 6913 were 5-ARIs naïve (age 66 years, IQR 60, 71; PSA 6.5 ng/ml, IQR 4.8, 9.0; prostate volume 50 ml, IQR 37, 65). MRI showed PIRADS 1–2, 3, 4, and 5 lesions in 141 (20%), 158 (22%), 258 (37%), and 148 (21%) patients treated with 5-ARIs, and 878 (13%), 1764 (25%), 2948 (43%), and 1323 (19%) of untreated patients (*p* < 0.0001). No difference was found in csPCa detection rates, but diagnosis of high-grade PCa (ISUP GG ≥ 3) was higher in treated patients (23% vs 19%, *p* = 0.013). We did not find any evidence of interaction between PIRADS score and 5-ARIs exposure in predicting csPCa. Sensitivity, specificity, PPV, and NPV of PIRADS ≥ 3 were 94%, 29%, 46%, and 88% in treated patients and 96%, 18%, 43%, and 88% in untreated patients, respectively.

**Conclusions:**

Exposure to 5-ARIs does not affect the association of PIRADS score with csPCa. Higher rates of high-grade PCa were detected in treated patients, but most were clearly visible on MRI as PIRADS 4 and 5 lesions.

**Trial registration:**

The present study was registered at ClinicalTrials.gov number: NCT05078359.

**Supplementary Information:**

The online version contains supplementary material available at 10.1007/s00345-023-04634-2.

## Introduction

5-Alpha-reductase inhibitors (5-ARIs) are widely used for treatment of bladder outlet obstruction symptoms secondary to benign prostatic hyperplasia. Prostate volume decreases by 25% after 3–6 months of treatment with 50% decrease in Prostate-specific antigen (PSA) levels [1]. Given the known association of androgens with the development of prostate cancer (PCa), two randomized controlled trials evaluating the chemopreventive effect of 5-ARIs showed a reduced incidence of low- and intermediate-risk PCa. However, a slight increase in Gleason group 4 and 5 PCa was found [2, 3], leading to a safety warning by the FDA in 2011 [4]. Subsequent analyses of PCPT data and another large population-based study showed no difference in all-cause mortality [5], and a 25%, non-statistically significant, reduction in PCa mortality [6].

Recently, these findings have been further supported by a large population-based study that found a decreased risk of death from PCa in men treated with 5-ARI for more than 6 years compared to men not treated with 5-ARI [7].

Taken together, the available evidence suggests that treatment with 5-ARIs might affect the accuracy of screening and diagnosis of PCa. The substantial decrease in serum prostate-specific antigen (PSA) levels potentially impact the predictive accuracy of PSA density (PSAd), risk calculator, and biomarkers [8–10]. On the other side, by reducing the prostate size, 5-ARIs might increase prostate biopsy (PBx) detection accuracy, leading to a potential detection bias that explains the increase incidence of high-risk PCa [11].

Magnetic Resonance imaging (MRI) of the prostate and MRI-targeted biopsies proved their outstanding diagnostic performance in the detection of PCa, with the delineation of the “MRI Pathway” [12] especially thanks to four landmark studies representing milestone in this field [13–16].

The MRI Pathway may help overcome the described limitations linked to treatment with 5-ARIs treatment, but on the other hand the use of 5‐ARIs is expected to induce significant phenotypic alterations in both benign prostatic hyperplasia (BPH) and PCa, potentially affecting the interpretation of MRI in patients treated with 5-ARIs [17].

The primary aim of the present study was to evaluate if exposure to 5-ARIs modifies the effect of MRI for the diagnosis of clinically significant PCa (csPCa). Therefore, we tried to assess the best biopsy strategy by combining PIRADS score and PSA density, both in untreated patients and patients treated with 5-ARI.

## Methods

### Study population: the PROMOD study

The PROstate Mri Outcome Database (PROMOD) study is a registered (ClinicalTrials.gov number, NCT05078359) retrospective observational study enrolling academic and non-academic institutions performing prostate biopsy. From January 2020 to January 2022, 36 institutions were invited to participate and submit individual patients’ datasets. Institutions were considered eligible if prostate MRI was performed prior to prostate biopsy according to PIRADS recommendations [18, 19]. Data for the present study were extracted from the PROMOD in May 2022.

Patients treated with 5-ARIs for at least 3 months at the time of MRI were included in the study group (treated), while 5-ARIs naïve patients were used as controls (untreated). Patients with a previous positive biopsy and patients who had undergone a short course of 5-ARIs or other surgical treatments for BPH were excluded. We did not use any adjustment factor to correct PSA values in treated patients. MRI-defined prostate volume was used to measure PSA density.

University of Foggia ethical committee approved the study protocol (143/CE/2020, DDG n. 696).

### MRI studies and biopsy techniques

Descriptions of the study cohorts, including MRI protocols and biopsy techniques are presented in supplementary material (Supplementary Table 1).

Data from two prospective clinical trials for the development of MRI imaging protocols (IMPROD NCT01864135, Multi-IMPROD NCT02241122) are included in the present study [20, 21].

An IMPROD bpMRI acquisition protocol (http://petiv.utu.fi/improd/) which consists of optimized T2-weighted (axial and sagittal) and three separate diffusion-weighted imaging (DWI) acquisitions was used in cohorts from Finland. All imaging datasets classified through five-tiered IMPROD bpMRI Likert scoring system were centrally reviewed by one reader and reported using PIRADS version 2.1 [22].

The decision to perform MRI was based on a clinical suspicion of PCa (positive digital rectal examination or elevated PSA levels). In case of negative MRI (PIRADS/Likert/IMPROD bpMRI Likert score < 3) all men received a 12- to 18-core standard systematic biopsy. If MRI was positive (i.e., PIRADS/Likert/IMPROD bpMRI Likert score ≥ 3), 2–4 extra cores were taken from each lesion (up to 4 lesions) through cognitive guidance or ultrasound/MRI fusion software. Our cohort included both biopsy-naïve patients and patients with a previous negative biopsy.

### Outcome measurements and statistical analysis

The primary outcome of the study was csPCa at PBx, defined as ISUP Gleason grade (ISUP GG) ≥ 2.

Statistical analyses have been performed using STATA 16 (StataCorp LLC, Texas, USA) through 4 consecutive steps.

First, descriptive statistics were obtained in patients untreated (control group) and treated (study group) with 5-ARIs. Continuous variables are reported as median and interquartile range (IQR) and were compared by the Mann–Whitney *U* test, whereas categorical variables are reported as rates and were tested by the Pearson Chi-square test, when appropriate.

Second, multivariable logistic regression analysis to predict CsPCa was performed including age at biopsy, digital rectal examination (DRE), biopsy history, PSA, prostate volume, PIRADS score, and exposure to 5-ARIs. An interaction term between 5-ARIs and PIRADS score was added to the model, and we graphed the probability of csPCa according to PIRADS in patients untreated and treated with 5-ARIs. Additionally, we calculated the linear combination of regression coefficients (STATA command: lincom, expressed as Odds Ratio) to demonstrate if PIRADS 3, 4, or 5 had a different association with csPCa in untreated vs treated group.

Third, we computed sensitivity, specificity, positive and negative predictive values, and accuracy of MRI for prediction of csPCa in the two study groups. As a sensitivity analysis we considered two definitions of positive MRI: (i) PIRADS/Likert score ≥ 3, and (ii) PIRADS/Likert score ≥ 4.

Finally, ten different biopsy strategies were simulated based on the combination of PSAd and MRI results. Decision curve analysis (DCA) for all proposed biopsy strategies was carried out to evaluate the best biopsy strategy for the detection of csPCa in treated and untreated men. Performing biopsy in all men, in no one, and performing biopsy based only on MRI findings were considered as reference strategies. The level of significance was set to 0.05.

## Results

### Study population baseline characteristics and cancer detection rates

The study flow chart with detailed number of patients excluded is presented in Supplementary Fig. 1. Out of 10,066 patients in the PROMOD database, 7618 patients from 24 institutions were ultimately eligible for the present study (Table 1). A total of 4403 (58%) patients were diagnosed with PCa, while 2994 (39.3%) were diagnosed with csPCa. Study group included 705 patients treated with 5-ARIs. Treated patients were older (69 vs 66, *p* < 0.0001) and had lower PSA values (6.0 vs 6.7, *p* 0.0001) and larger prostate volumes (53 vs 50, *p* < 0.0001). Prostate MRI showed PIRADS 1–2, 3, 4, and 5 lesions in 141 (20%), 158 (22%), 258 (37%), and 148 (21%) treated patients, and 878 (13%), 1764 (25%), 2948 (43%), and 1323 (19%) of untreated patients (*p* < 0.0001). Central zone and transition zone lesions were more frequent in the treated group (19% vs 23%, *p* 0.01).

**Table 1:** Descriptive characteristics of the study population

	Overall population (*N* = 7618)	5-ARI untreated (*N* = 6913)	5-ARI treated (*N* = 705)	*p* value
Age (year)	66 (60, 71)	66 (60, 71)	69 (65, 73)	< 0.0001
Previous biopsy history, *n* (%)				
Biopsy Naive	6074 (79.7%)	5555 (80.4%)	519 (73.6%)	< 0.0001
Previous negative	1544 (20.3%)	1358 (19.6%)	186 (26.4%)	
DRE, *n* (%)				
Negative	5413 (71.1%)	4887 (70.7%)	526 (74.6%)	0.029
Suspicious	2205 (28.9%)	2026 (29.3%)	179 (25.4%)	
PSA, ng/ml	6.5 (4.7, 9.0)	6.5 (4.8, 9.0)	6.0 (4.0, 9.0)	0.0001
Prostate volume, ml	50 (38, 65)	50 (37, 65)	53 (40, 72)	< 0.0001
PSA density	0.13 (0.09, 0.19)	0.13 (0.09, 0.19)	0.11 (0.07, 0.18)	< 0.0001
PIRADS, *n* (%)				
1–2	1019 (13.4%)	878 (12.7%)	141 (20.0%)	< 0.0001
3	1922 (25.2%)	1764 (25.5%)	158 (22.4%)	
4	3206 (42.1%)	2948 (42.6%)	258 (36.6%)	
5	1471 (19.3%)	1323 (19.1%)	148 (21.0%)	
Index lesion loc, *n* (%)^a^				
PZ	5346 (81.0%)	4912 (81.4%)	434 (77.0%)	0.010
CZ-TZ	1253 (19.0%)	1123 (18.6%)	130 (23.0%)	
Index lesion volume, ml	0.52 (0.27, 1.37)	0.52 (0.27, 1.29)	0.69 (0.27, 1.77)	0.001
Biopsy ISUP GG, *n* (%)				
Negative	3215 (42.2%)	2900 (41.9%)	315 (44.7%)	< 0.0001
1	1409 (18.5%)	1295 (18.7%)	114 (16.2%)	
2	1533 (20.1%)	1417 (20.5%)	116 (16.5%)	
3	673 (8.8%)	608 (8.8%)	65 (9.2%)	
4	515 (6.8%)	468 (6.8%)	47 (6.7%)	
5	273 (3.6%)	225 (3.3%)	48 (6.8%)	
ISUP GG ≥ 2, *n* (%)	2994 (39.3%)	2718 (39.3%)	276 (39.1%)	0.9
ISUP GG ≥ 3, *n* (%)	1461 (19.2%)	1301 (18.8%)	160 (22.7%)	0.013

^a^Computed on the number of patients with a positive MRI (PIRADS > 2)

There was no significant difference in csPCa (ISUP GG ≥ 2) detection rates (39% vs 39%, *p* 0.9); however, the detection of high-grade PCa (ISUP GG ≥ 3) was significantly higher in treated patients (19% vs 23%, *p* 0.013).

### Multivariable logistic regression and interaction analysis

At multivariable logistic regression analysis, treatment with 5-ARIs (included as covariate) was not found to be associated with diagnosis of csPCa (OR 1.05, CI 0.6, 1.84; *p* 0.876) (Supplementary table 2). The probability of csPCa according to PIRADS score was similar in patients untreated and treated with 5-ARIs (Supplementary Fig. 2). Similarly, untreated and treated patients have similar csPCa regression coefficients for PIRADS 3 (OR 1.01; CI 0.94, 1.08; *p* 0.846), PIRADS 4 (OR 1.02; CI 0.96, 1.08; *p* 0.532), and PIRADS 5 (OR 1.06; CI 0.98, 1.15; *p* 0.124).

### Accuracy of prostate MRI

**Fig. 1:**
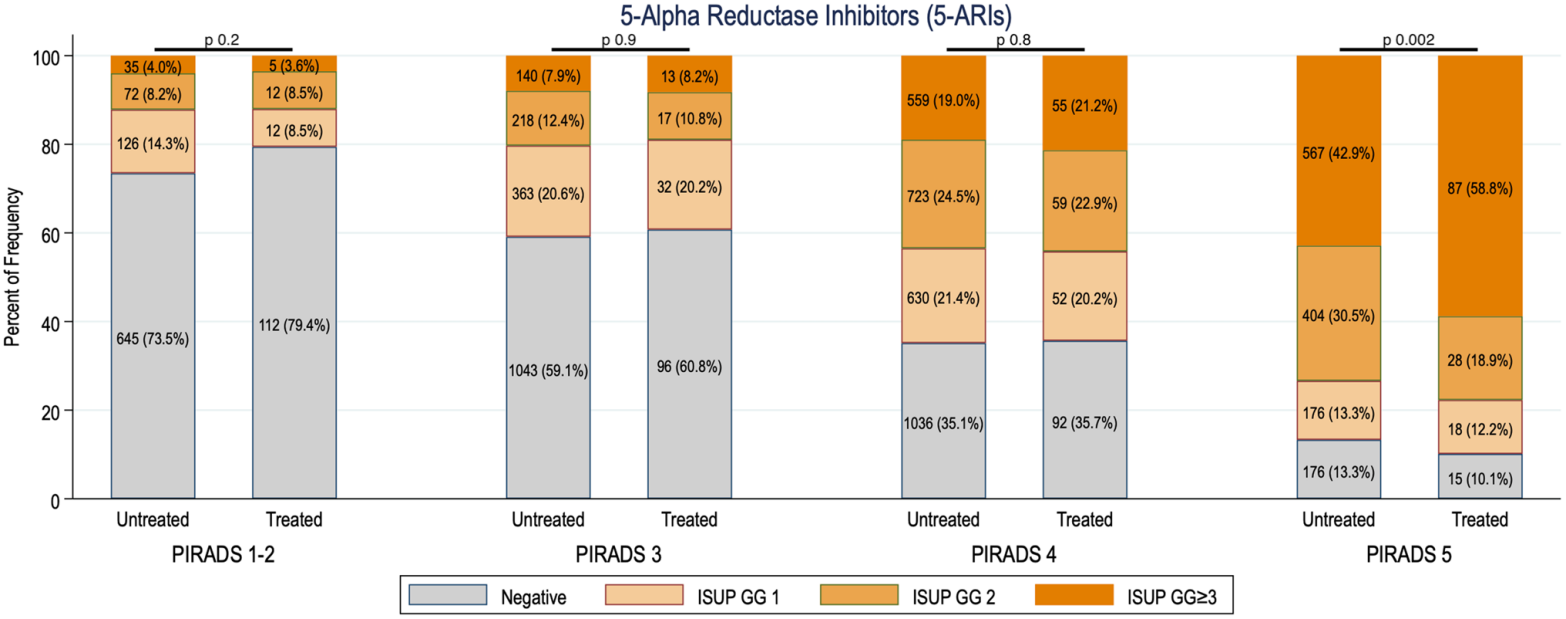
Prostate cancer detection rates of patients untreated and treated with 5-ARI’s according to PIRADS score. Detection rates of each PIRADS score were compared between the two groups: no difference was found in biopsy results in PIRADS 1–2 (*p* 0.2), PIRADS 3 (*p* 0.9), and PIRADS 4 (*p* 0.8). Conversely cancer detection rates were higher in PIRADS 5 patients treated with 5-ARI’s (*p* 0.002)

Prostate cancer detection rates by PIRADS score were compared between the two groups and no difference was found in biopsy results in PIRADS 1–2 (*p* 0.2), PIRADS 3 (*p* 0.9), and PIRADS 4 (*p* 0.8). Conversely cancer detection rates were higher in PIRADS 5 treated patients (*p* 0.002, Fig. 1). The accuracy of MRI was similar in the two groups. Specifically, with a definition of positive MRI as PIRADS/Likert score ≥ 3, sensitivity, specificity, PPV, and NPV for csPCa were 94%, 29%, 46%, and 88% in treated patients and 96%, 18%, 43%, and 88% in untreated patients, respectively (Supplementary Table 3).

In patients treated with 5-ARIs, 276 csPCas were diagnosed. Of these, 30 (10.9%) were visible as PIRADS 3 lesion on MRI and 229 (83.0%) as PIRADS 4–5 lesions. Similarly, high-grade PCa (ISUP GG ≥ 3, *n* = 160) was visible as PIRADS 3 lesion in 13 (8.1%) patients and PIRADS 4–5 lesions in 142 (88.8%) patients (Supplementary Table 4).

Representative clinical MRI images of a patient treated with dutasteride are presented in Supplementary Fig. 3.

### Best diagnostic strategies in patients treated with 5-ARI

According to DCA (Supplementary Fig. 4A), the best diagnostic strategies in untreated patients were #7 (PIRADS/Likert 4–5 or PIRADS 3 if PSAd > 0.2), #8 (PIRADS/Likert 4–5 or PIRADS/Likert 3 if PSAd > 0.15), and #1 (PIRADS/Likert 4–5 or PSAd > 0.2) resulting in ~ 30% of biopsy avoidance, 24–30% reduction in the diagnosis of GGG 1 PCa while missing ~ 10% of csPCa. In treated patients (Supplementary Fig. 4B), strategy #7 led to the highest net benefit at decision curve analysis. This would lead to 40% in biopsy avoidance, 35% reduction in GG 1 PCa diagnosis, and 15% of csPCa missed. Similarly, the second-best biopsy strategy according to DCA was #8, with a slightly lower number of csPCa missed (12%). Number of biopsies avoided, GG 1 PCa diagnosis and csPCa missed in patients treated and untreated are presented for all the strategies in Supplementary Table 5.

## Discussion

In this large, multicenter cohort of patients who had undergone MRI for suspicion of PCa, we sought to determine the impact of exposure to 5-ARIs on the ability of MRI to predict biopsy outcomes.

Our results show that MRI had similar diagnostic accuracy for prebiopsy risk stratification in both the study groups. Furthermore, we found a slightly higher rate of high-grade PCa in treated patients, most of them were visible on MRI as PIRADS 4 and 5 lesions. These findings corroborate previously published results on the performance of MRI in this subset of patients, here represented by the largest cohort investigated by far [23, 24]. Although there were no significant differences in lesion size or lesion volume for MRI-visible PCa lesions, in patients treated with 5-ARIs, ADC metrics (e.g., ADC lesion values, ADC lesion/benign and ADC lesion/urine ratios) were less effective in the distinction between csPCa and non-csPCa or benign lesions [24]. Similarly, Giganti et al. randomized 37 men with a previous diagnosis of low-grade PCa, to 6 months of dutasteride or placebo [25] and subsequently evaluated the MRI changes in ADC values and T2W imaging [26, 27]. While the exposure to antiandrogen therapy did not significantly influence the T2 contrast or the T2 relaxation values [27], the absolute changes in ADC and conspicuity varied significantly between the two groups at 6 months [26]. We were not able to perform per lesion analysis using ADC metrics; however, even assuming a lower accuracy of ADC metrics after exposure to 5-ARIs, this does not translate into different accuracy in the assessment of PIRADS score.

Evidence from the PCPT showed that PSA had better sensitivity and AUC for detecting PCa in patients treated with finasteride for more than 1 year compared to untreated patients [8]. However, in men treated with 5-ARIs for more than 1 year, time varying adjustment factors (from 2 at 24 months to 2.5 at 7 years after the initiation of finasteride) are needed to determine whether PSA is in the normal range [28].

The evidence on the impact of shorter-term treatment with 5-ARIs is scarce; however, 3–6 months after the start of therapy, most of the phenotypic changes in the tissue have happened with a consequent reduction of PSA to its minimal levels or very close to them. Most studies on the accuracy of biomarkers for PCa excluded treated patients or did not report if patients were taking this class of medication [29]. However, preliminary results from a randomized trial reported a significant effect of the treatment on biomarker values, suggesting that these results should be interpreted with caution in patients receiving finasteride until formal validation of test performance in these patients is conducted [30].

The importance of PSA density in association with MRI results for the diagnosis of PCa was confirmed by our study, where the best strategies to submit patients to PBx were similar in the two study groups, regardless of 5-ARI treatment.

To the best of our knowledge, this is the first multicenter and the largest study testing the interaction of exposure to 5-ARIs with PIRADS score for the diagnosis of csPCa, and our findings must be viewed in light of two main factors. First, MRI is a pivotal step in the novel screening algorithms and 5-ARIs are used by up to 10% of the general population. Second, the evidence supporting the positive effect of 5-ARIs on PCa mortality is growing and, with the introduction of MRI in the diagnostic pathway, the effect of such medications might be increasingly evident in upcoming years.

On the other side, we recognize a few limitations of this report. This is a retrospective study on patients undergoing biopsy with prebiopsy MRI and no long-term follow-up was available to evaluate oncological outcomes of PCa diagnosed by MRI in treated patients. No information was available on the duration of treatment with 5-ARIs (only recorded as ≥ 3 months). This precludes us from drawing any conclusion on the development of high-grade PCa following 5-ARIs exposure. This was beyond the scope of the present study, and we believe that the accuracy of MRI in the diagnosis of PCa does not change over time of exposure. Additionally, we excluded all patients with treatment duration of less than 3 months to ensure a full prostatic response to the therapy. Most changes in PSA and prostate volume occur within 3 months and therefore by extension this study would capture any changes on prostate MRI. Finally, our study included patients undergoing biopsy and MRI in 24 different institutions with wide variations in MRI acquisition protocols, following both PIRADS v2 and v2.1 recommendations, MRI scanners, biopsy techniques, and level of expertise of radiologists and urologists [31]. While this is a limitation in the absence of central MRI and pathology reporting, we believe it represents also one of the main strengths of our study that provide a picture of the accuracy of MRI in academic and non-academic centers.

## Conclusion

Exposure to 5-ARIs does not affect the efficacy of PIRADS score for prebiopsy risk stratification in patients who underwent treatment with 5-ARIs and who did not. The MRI diagnostic pathway can be safely used in patients treated with 5-ARIs and detects a comparable amount of cancer compared to 5-ARIs naïve patients.

### Supplementary Information

Below is the link to the electronic supplementary material.

Supplementary file1 (DOCX 24930 KB)

## Data Availability

Derived data supporting the findings of this study are available from the corresponding author UGF on request.
