# Supplementary material for: Diagnosis of prostate cancer with magnetic resonance imaging in men treated with 5-alpha-reductase inhibitors
Source: World J Urol. 2023 Oct 3;41(11):2967–74. doi: 10.1007/s00345-023-04634-2 (PMC10632288; doi:10.1007/s00345-023-04634-2)

**Supplementary Table 1.** Biopsy and MRI techniques of the institutions participating in the PROMOD study.

| **Intitution #** | **Patients enrolled** | **Country** | **Setting** | **Operator** | **MRI Tech** | **Radiologists involved** | **Radiologist years of Experience** | **MRI reporting system** | **technique of target biopsy** | **Route** | **target cores per lesion** | **Systematic cores** |
| --- | --- | --- | --- | --- | --- | --- | --- | --- | --- | --- | --- | --- |
| 1 | 1356 | Italy | Academic | Urologist | 3 Tesla Multiparametric MRI (with contrast agent) | 3 | 10 | PIRADS | MRI-US Fusion/ In bore MRI | Transperineal | 5 | 12 |
| 2 | 922 | Italy | Academic | Urologist | 1,5 Tesla Multiparametric MRI (with contrast agent) | 2 | 6 | PIRADS | MRI-US Fusion | Transrectal | 2 | 14 |
| 3 | 798 | Italy | Academic | Urologist | 1,5 Tesla Multiparametric MRI (with contrast agent) | 3 | 5 | PIRADS | MRI-US Fusion | Transperineal | 4 | 12 |
| 4 | 727 | Italy | Academic | Urologist | 3 Tesla Multiparametric MRI (with contrast agent) | 2 | 4 | PIRADS | MRI-US Fusion | Transperineal | 3 | >14 |
| 5 | 671 | Italy | Academic | Urologist | 1,5 Tesla Multiparametric MRI (with contrast agent) | 3 | 8 | PIRADS | MRI-US Fusion / cognitive | Transrectal | 6 | 12 |
| 6 | 543 | Italy | Academic | Urologist | 1,5 Tesla Multiparametric MRI (with contrast agent) | 8 | 10 | PIRADS | MRI-US cognitive | Transperineal | 2 | 14 |
| 7 | 531 | Sweden-Norwey | Academic | Urologist | 1,5 Tesla Multiparametric MRI (with contrast agent) |  |  | PIRADS | MRI-US Fusion | Transrectal | 3 | 12 |
| 8 | 483 | Italy | Academic | Urologist | 1,5 Tesla Multiparametric MRI (with contrast agent) | 4 | 8 | PIRADS | MRI-US Fusion | Transperineal | 3 | 12 |
| 9 | 805 | Finland | Academic | Urologist | 3 Tesla Biparametric MRI (no contrast agent) | 1 | 8 | IMPROD Likert | MRI-US cognitive | Transrectal | 2 | 12 |
| 10 | 396 | Italy | Academic | Urologist | 3 Tesla Multiparametric MRI (with contrast agent) | 4 | 2 | PIRADS | MRI-US Fusion | Transrectal | 2 | 12 |
| 11 | 392 | Italy | Non academic public hospital | Urologist | 3 Tesla Multiparametric MRI (with contrast agent) | 1 | 15 | PIRADS | MRI-US Fusion | Transperineal | 3 | >14 |
| 12 | 275 | Italy | Academic | Urologist | 3 Tesla Multiparametric MRI (with contrast agent) | 2 | 8 | PIRADS | MRI-US Fusion / cognitive | Transrectal | 4 | 12 |
| 13 | 209 | Italy | Academic | Urologist | 1,5 Tesla Multiparametric MRI (with contrast agent) | 2 | 5 | PIRADS | MRI-US Fusion / cognitive | Transrectal | 3 | 12 |
| 14 | 383 | Italy | Non academic public hospital | Urologist | 1,5 Tesla Multiparametric MRI (with contrast agent) | 1 | 6 | PIRADS | MRI-US Fusion / cognitive | Transperineal | 3 | 12 |
| 15 | 186 | Italy | Academic | Urologist | 3 Tesla Multiparametric MRI (with contrast agent) | 2 | 25 | PIRADS | MRI-US cognitive | Transrectal | 3 | 12 |
| 16 | 381 | Italy | Academic | Urologist | 1,5 Tesla Multiparametric MRI (with contrast agent) | 1 | 5 | PIRADS | MRI-US Fusion | Transperineal | 3 | 14 |
| 17 | 182 | Italy | Academic | Radiologist | 3 Tesla Multiparametric MRI (with contrast agent) | 1 | 10 | PIRADS | MRI-US Fusion/ In bore MRI | Transperineal | 3 | 12 |
| 18 | 177 | Italy | Private practice | Urologist | 1,5 Tesla Multiparametric MRI (with contrast agent) | 5 | 10 | PIRADS | MRI-US Fusion | Transperineal | 2 | 12 |
| 19 | 109 | Italy | Academic | Urologist | 1,5 Tesla Multiparametric MRI (with contrast agent) | 1 | 5 | PIRADS | MRI-US Fusion | Transperineal | 3 | 14 |
| 20 | 164 | Finland | Academic | Urologist | 1,5 Tesla Biparametric MRI (no contrast agent) | 1 | 8 | IMPROD Likert | MRI-US cognitive | Transrectal | 2 | 12 |
| 21 | 59 | Finland | Academic | Urologist | 3 Tesla Biparametric MRI (no contrast agent) | 1 | 8 | IMPROD Likert | MRI-US Fusion | Transrectal | 2 | 12 |
| 22 | 155 | Finland | Academic | Urologist | 3 Tesla Biparametric MRI (no contrast agent) | 1 | 8 | IMPROD Likert | MRI-US cognitive | Transrectal | 2 | 12 |
| 23 | 45 | Italy | Non academic public hospital | Urologist | 1,5 Tesla Biparametric MRI (no contrast agent) | 5 | 5 | PIRADS | MRI-US Fusion | Transperineal | 2 | >14 |
| 24 | 117 | Finland | Non academic public hospital | Urologist | 1,5 Tesla Multiparametric MRI (with contrast agent) | 1 | 8 | IMPROD Likert | MRI-US cognitive | Transrectal | 3 | >14 |

**Supplementary Table 2.** Multivariable Logistic regression analysis to predict CsPCa.

|  | **MM predicting csPCa**  **(ISUP GG≥2)** | | |
| --- | --- | --- | --- |
|  | **OR** | **95% CI** | **P>\|z\|** |
| **Age** |  |  |  |
| per year | 1.04 | 1.04,1.05 | <0.001 |
| **Biopsy History** |  |  |  |
| Naive | Ref. |  |  |
| Previous Neg | 0.60 | 0.52,0.69 | <0.001 |
| **DRE** |  |  |  |
| Negative | Ref. |  |  |
| Suspicious | 1.90 | 1.69,2.14 | <0.001 |
| **PSA** |  |  |  |
| per ng/ml | 1.11 | 1.09,1.12 | <0.001 |
| **MRI Volume** |  |  |  |
| per cc | 0.98 | 0.97,0.98 | <0.001 |
| **PIRADS** |  |  |  |
| 1-2 | Ref. |  |  |
| 3 | 1.70 | 1.34,2.17 | <0.001 |
| 4 | 4.58 | 3.66,5.73 | <0.001 |
| 5 | 12.64 | 9.87,16.19 | <0.001 |
| **5 ARI** |  |  |  |
| Untreated | Ref. |  |  |
| Treated | 1.05 | 0.6,1.84 | 0.876 |
| **PIRADS#5ARI** |  |  |  |
| 3 (#5ARI treated) | 1.00 | 0.49,2.03 | 0.996 |
| 4 (#5ARI treated) | 1.04 | 0.56,1.96 | 0.892 |
| 5 (#5ARI treated) | 1.33 | 0.65,2.69 | 0.437 |

**Supplementary Table 3.** Sensitivity, specificity, positive and negative predictive values and Accuracy of MRI for prediction of csPCa in the overall cohort of 5-ARI treated and untreated patients undergoing prostate biopsy.

|  | **5-ARI Untreated Patients** | | **5-ARI Treated Patients** | |
| --- | --- | --- | --- | --- |
|  | **Value** | **95% CI** | **Value** | **95% CI** |
| **Positive MRI (PIRADS≥3)** |  |  |  |  |
| **Sensitivity** | 96.06% | 95.26, 96.76 | 93.84% | 90.32, 96.37 |
| **Specificity** | 18.38% | 17.22, 19.58 | 28.90% | 24.66, 33.45 |
| **Positive Predictive Value (*)** | 43.26% | 42.87, 43.66 | 45.92% | 44.25, 47.60 |
| **Negative Predictive Value (*)** | 87.81% | 85.55, 89.76 | 87.94% | 81.81, 92.21 |
| **Accuracy (*)** | 48.92% | 47.74, 50.11 | 54.33% | 50.57, 58.05 |
| **Positive MRI (PIRADS≥4)** |  |  |  |  |
| **Sensitivity** | 82.89% | 81.42, 84.29 | 82.97% | 78.01, 87.21 |
| **Specificity** | 51.90% | 50.37, 53.42 | 58.74% | 53.92, 63.44 |
| **Positive Predictive Value (*)** | 52.75% | 51.86, 53.64 | 56.40% | 53.31, 59.45 |
| **Negative Predictive Value (*)** | 82.40% | 81.09, 83.64 | 84.28% | 80.33, 87.56 |
| **Accuracy (*)** | 64.08% | 62.94, 65.21 | 68.23% | 64.65, 71.65 |
| (*) These values are dependent on disease prevalence | | | | |

**Supplementary Table 4.** MRI Findings in patients with csPCa and High grade PCa treated and untreated with 5-ARI

|  | **Cs PCa (ISUP GG≥2)** | |  | **High Grade PCa (ISUP GG≥3)** | |  |
| --- | --- | --- | --- | --- | --- | --- |
|  | **5-ARI Untreated (N=2718)** | **5-ARI Treated (N=276)** | **P value** | **5-ARI Untreated (N=1301)** | **5-ARI Treated (N=160)** | **P value** |
| **GG Bx, n (%)** |  |  |  |  |  |  |
| 2 | 1417 (52.1%) | 116 (42.0%) | **<0.0001** | N/A | N/A | 0.0005 |
| 3 | 608 (22.4%) | 65 (23.6%) |  | 608 (46.7%) | 65 (40.6%) |  |
| 4 | 468 (17.2%) | 47 (17.0%) |  | 468 (36.0%) | 47 (29.4%) |  |
| 5 | 225 (8.3%) | 48 (17.4%) |  | 225 (17.3%) | 48 (30.0%) |  |
| **PIRADS, n (%)** |  |  |  |  |  |  |
| 1-2 | 107 (3.9%) | 17 (6.2%) | **0.04** | 35 (2.7%) | 5 (3.1%) | 0.064 |
| 3 | 358 (13.2%) | 30 (10.9%) |  | 140 (10.8%) | 13 (8.1%) |  |
| 4 | 1282 (47.2%) | 114 (41.3%) |  | 559 (43.0%) | 55 (34.4%) |  |
| 5 | 971 (35.7%) | 115 (41.7%) |  | 567 (43.6%) | 87 (54.4%) |  |
| **Index Lesion Location, n (%)** |  |  |  |  |  |  |
| No Lesion | 107 (3.9%) | 17 (6.2%) | 0.094 | 35 (2.7%) | 5 (3.1%) | 0.7 |
| PZ | 2244 (82.6%) | 215 (77.9%) |  | 1099 (84.5%) | 131 (81.9%) |  |
| CZ-TZ | 367 (13.5%) | 44 (15.9%) |  | 167 (12.8%) | 24 (15.0%) |  |
| **Index Lesion Volume, cc** | 0.69 (0.27, 1.77) | 0.75 (0.27, 1.77) | 0.4 | 0.90 (0.38, 2.28) | 0.90 (0.38, 2.10) | 0.8 |

**Supplementary Table 5. Summary table of ten different strategy including MRI and PSA density parameters to assess the need for prostate biopsy.**

The number of Biopsy avoided, ISUP Gleason Group (GG) 1 and GG ≥2 Cancers missed are shown for each strategy in 5-ARI treated and untreated patients. Biopsy decision based on MRI alone was used as reference (Ref). In untreated patients setting strategy 1, strategy 7, and strategy 8 showed the best balance of biopsy avoided and ISUP GG ≥2 missed with the lowest % of GG 1 diagnosed. Similar results but higher rates of biopsy avoidance were found in treated patients.

|  |  | **5-ARI Untreated** | | | **5-ARI Treated** | | |
| --- | --- | --- | --- | --- | --- | --- | --- |
| **#** | **Strategy description** | **Biopsy avoided** | **GGG 1 missed** | **GGG ≥2 Missed** | **Biopsy avoided** | **GGG 1 missed** | **GGG ≥2 Missed** |
|  | **Biopsy if…** | N (%) | N (%) | N (%) | N (%) | N (%) | N (%) |
| Ref | PI-RADS 3-4-5 | 878 (12.7) | 126 (9.7) | 107 (3.9) | 141 (20) | 12 (10.5) | 17 (6.2) |
| 1 | PI-RADS 4-5 or PSAd>0.2 | 2252 (32.6) | 414 (32) | 340 (12.5) | 265 (37.6) | 40 (35.1) | 38 (13.8) |
| 2 | PI-RADS 4-5 or PSAd>0.15 | 1847 (26.7) | 320 (24.7) | 254 (9.3) | 233 (33) | 36 (31.6) | 29 (10.5) |
| 3 | PI-RADS 4-5 or PSAd>0.10 | 1073 (15.5) | 173 (13.4) | 116 (4.3) | 163 (23.1) | 27 (23.7) | 18 (6.5) |
| 4 | PI-RADS 3-4-5 or PSAd>0.2 | 764 (11.1) | 100 (7.7) | 83 (3.1) | 125 (17.1) | 12 (10.5) | 14 (5.1) |
| 5 | PI-RADS 3-4-5 or PSAd>0.15 | 632 (9.1) | 76 (5.9) | 61 (2.2) | 113 (16) | 11 (9.6) | 12 (4.3) |
| 6 | PI-RADS 3-4-5 or PSAd>0.10 | 381 (5.5) | 45 (3.5) | 30 (1.1) | 77 (10.9) | 8 (7) | 7 (2.5) |
| 7 | PI-RADS 4-5 or PIRADS 3 if PSAd>0.2 | 2366 (34.2) | 440 (34) | 364 (13.4) | 281 (39.9) | 40 (35.1) | 41 (14.9) |
| 8 | PI-RADS 4-5 or PIRADS 3 if PSAd>0.15 | 2093 (30.3) | 370 (28.6) | 300 (11) | 261 (37) | 37 (32.5) | 34 (12.3) |
| 9 | PI-RADS 4-5 or PIRADS 3 if PSAd>0.10 | 1570 (22.7) | 254 (19.6) | 193 (7.1) | 227 (32.2) | 31 (27.2) | 28 (10.1) |
| 10 | PI-RADS 4-5 or PIRADS 3 if PSAd>0.10 or PSAd>0.2 | 1456 (21.1) | 228 (17.6) | 169 (6.2) | 211 (29.9) | 31 (27.2) | 25 (9.1) |

**Supplementary Figure 1.** Study Flow Chart.

**Supplementary Figure 2.** Probability of csPCa according to PIRADS in patients untreated and treated with 5ARIs.


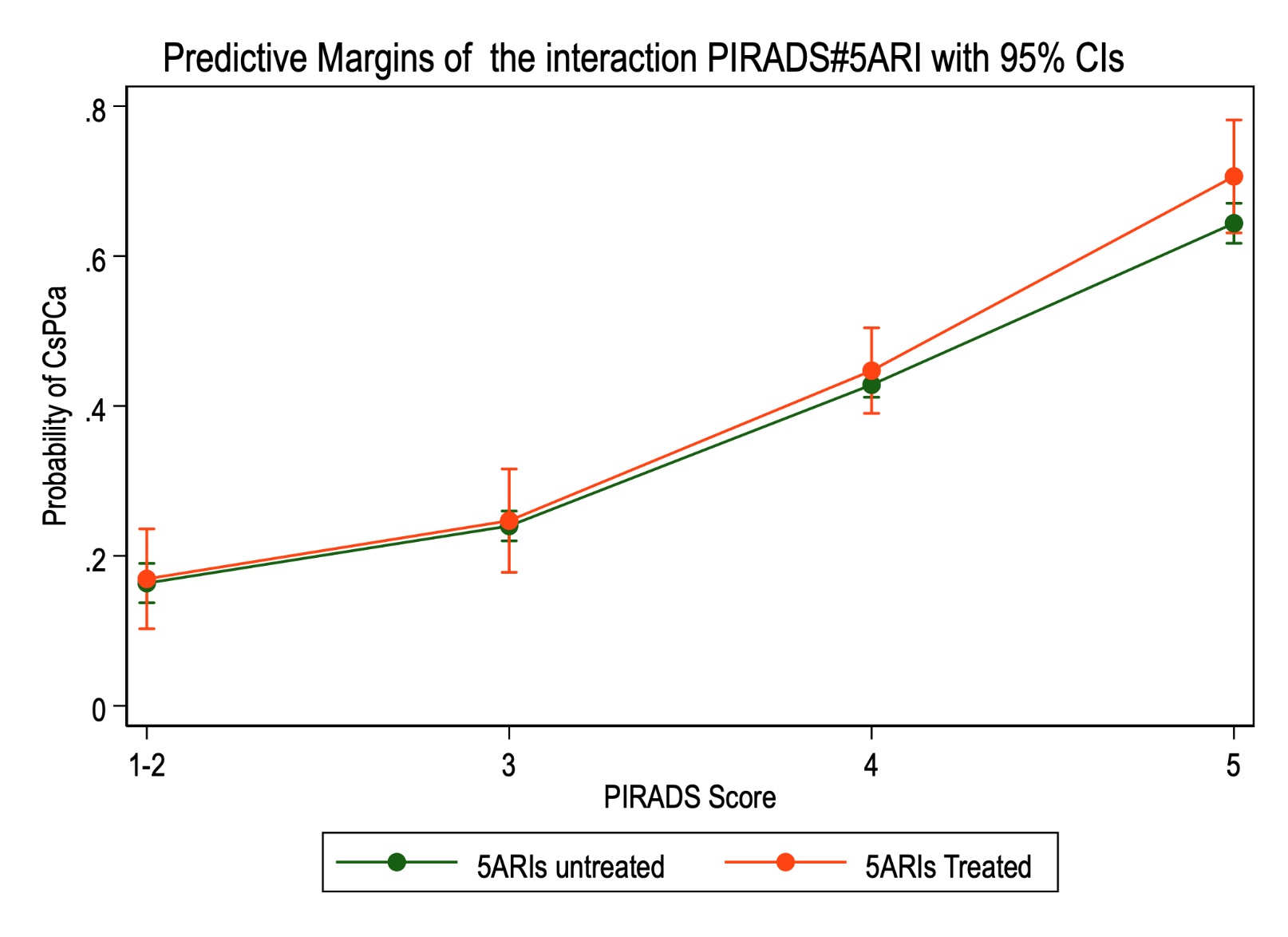


**Supplementary Figure 3.** **Representative clinical MRI images of a patient treated with dutasteride.** 71-year-old man with clinical suspicion of PCa (PSA total value of 14.4 ng/ml), previous negative systematic biopsy, treated with dutasteride for two years. (a) T2WI acquired on the axial plane showing a hypointense nodular lesion on the mid-right posterior-lateral zone, showing early and marked post-contrast enhancement on DCE images (b), with restricted diffusion on DWI at b-value 2000 (c) and low ADC value (d), classified as PI-RADS 4. The nodule was biopsied using MRI-TRUS TBx and histopathology confirmed the presence csPCa, ISUP 3 (GS 4 + 3). PCa, Prostate cancer; PSA, Prostate-specific antigen; T2WI, T2-weighted imaging; DCE, Dynamic contrast enhanced; DWI, Diffusion weighted imaging; ADC, Apparent diffusion coefficient; TBx, Targeted biopsy; ISUP, International society of urogenital pathology; GS, Gleason score.


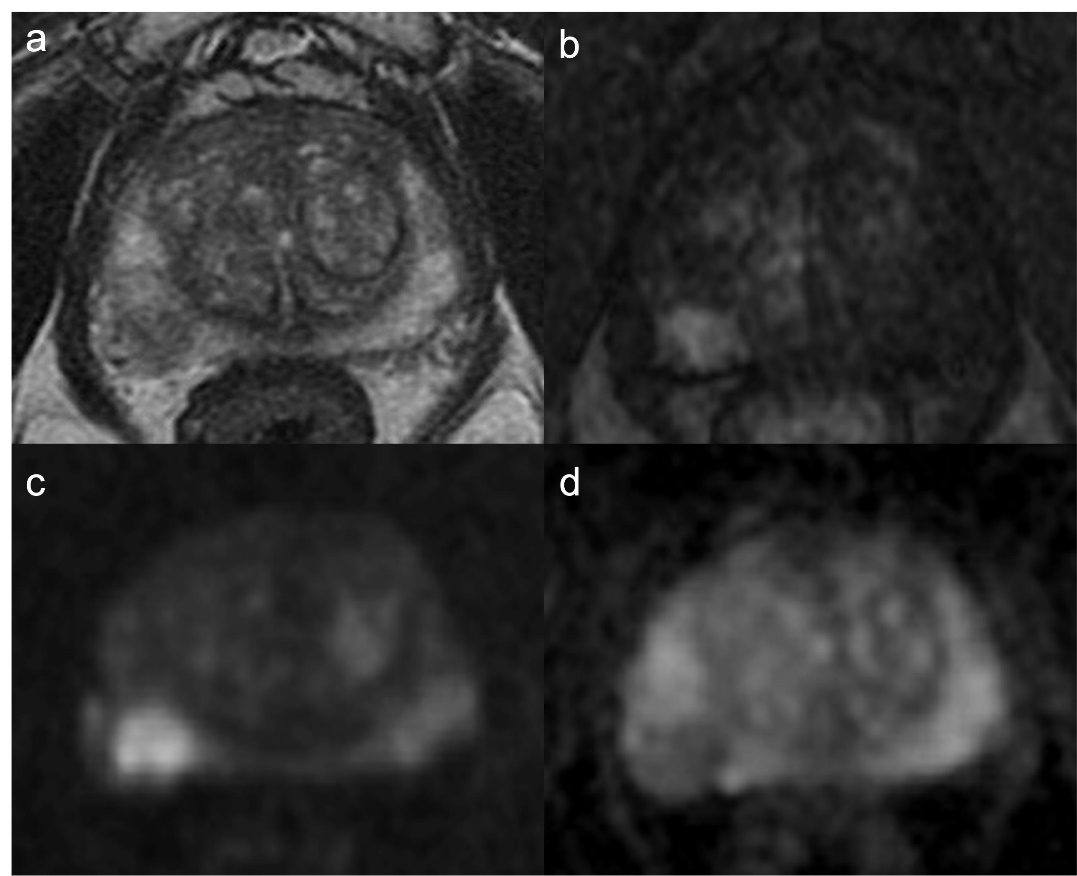


**Supplementary Figure 4.** **Decision curve analysis (DCA) comparing clinical utility of different biopsy strategy for detecting clinically significant prostate cancer in 5-ARI untreated (A) and treated patients (B).** The DCA simulates two scenarios: one in which all patients would receive biopsy (all) and one in which none undergoes biopsy (none). Clinically useful models lie above these scenarios. The graph gives the expected net benefit per patient relative to biopsy none. The unit is the benefit associated with one PCa patient duly undergoing biopsy.

Ref: PI- RADS/Likert 3-4-5

Strategy 1: PI-RADS/Likert 4-5 or PSAd>0.2

Strategy 2: PI-RADS/Likert 4-5 or PSAd>0.15

Strategy 3: PI-RADS/Likert 4-5 or PSAd>0.10

Strategy 4: PI-RADS/Likert 3-4-5 or PSAd>0.2

Strategy 5: PI-RADS/Likert 3-4-5 or PSAd>0.15

Strategy 6: PI-RADS/Likert 3-4-5 or PSAd>0.10

Strategy 7: PI-RADS/Likert 4-5 or PI-RADS/Likert 3 if PSAd>0.2

Strategy 8: PI-RADS/Likert 4-5 or PI-RADS/Likert 3 if PSAd>0.15

Strategy 9: PI-RADS/Likert 4-5 or PI-RADS 3 if PSAd>0.10

Strategy 10: PI-RADS/Likert 4-5 or PI-RADS/Likert 3 if PSAd>0.10 or PSAd>0.2


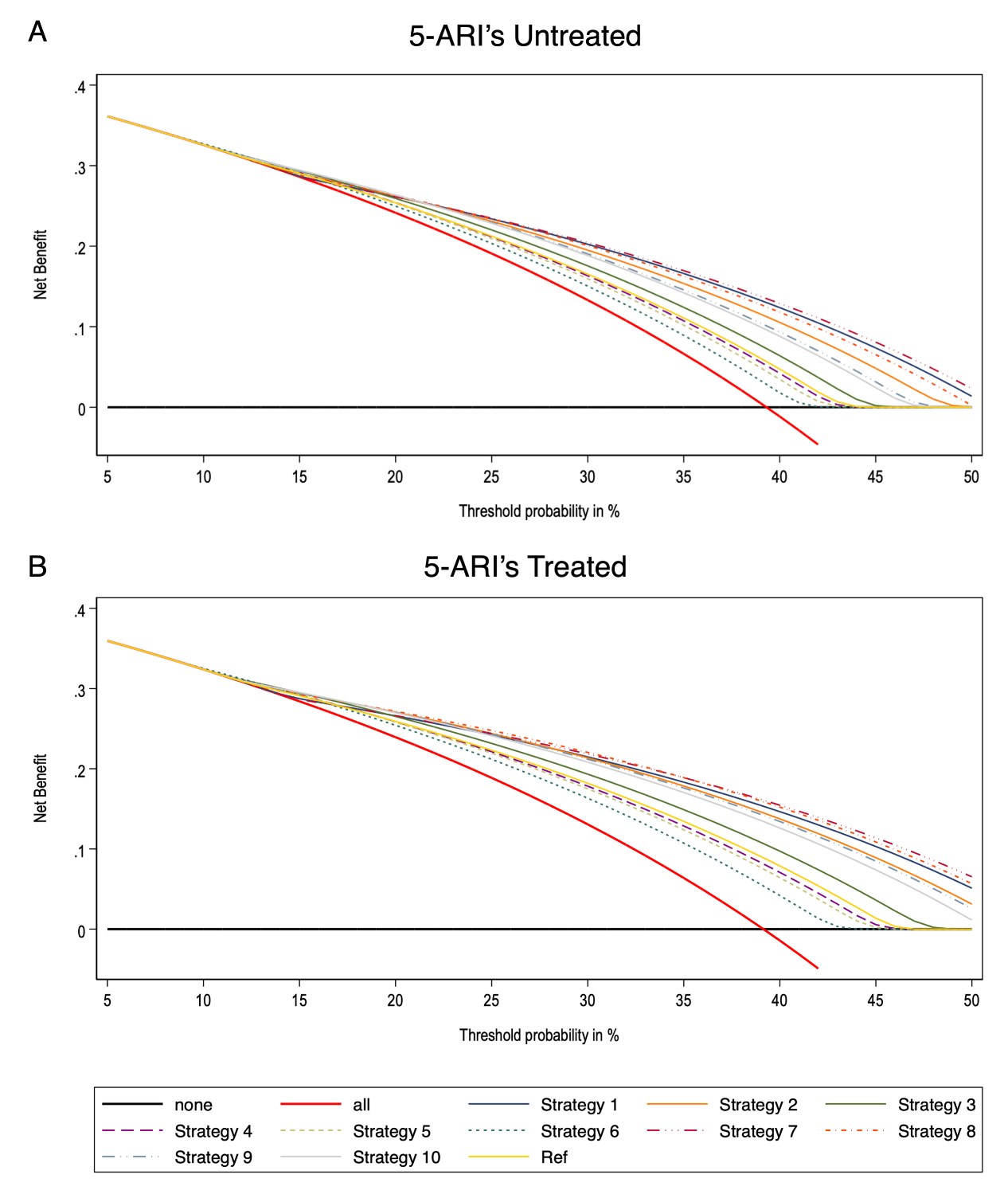

Supplement: Supplementary file 1 — Supplementary file1 (DOCX 24930 KB) [file 345_2023_4634_MOESM1_ESM.docx]
